# Assessment of HIV discordance and associated risk factors among couples receiving HIV test in Dilla, Ethiopia

**DOI:** 10.1186/1756-0500-7-893

**Published:** 2014-12-10

**Authors:** Moges Tadesse

**Affiliations:** Dilla University, College of health sciences and medicine, Dilla, Ethiopia

**Keywords:** HIV, Discordant, Couples, Dilla, Ethiopia

## Abstract

**Background:**

About 60% of new HIV infections occur in HIV sero-discordant couples as 30% of married HIV positives have negative spouse. Although HIV-discordant couples are at 10% annual risk of acquiring HIV infection and large number of new HIV infections occur in stable partnerships, most HIV prevention programs focus on reducing casual sexual partners, condom use, and increasing fidelity among married partners. The purpose is assessment of sero-discordance among couples and associated factors.

**Methods:**

The study was facility based cross sectional survey of couples who tested for HIV in 2011 and lived together at least 1 year period. The sample size was 154 couples (308 individuals) and necessary ethical issues were considered.

**Results:**

Among 152 couples (304 individuals) who received VCT, HIV sero-prevalence in this study was found to be 11(3.6%). The prevalence in females 8(5.3%) was higher than that in males 3(2.0%). Of all participants, 9(3.0%) were found to be sero-discordant, 2(0.7%) concordant positive and 293(96.4%) concordant negative. Of all couples 9(5.9%) were found to be sero-discordant, 2(1.3%) concordant positive and 141(92.8%) concordant negative. Of the 9 sero-discordant couples, the 7(4.6%) prevalence in females was higher than 2(1.3%) in males. And, among 9 discordant couples, 5 of them were premarital sex partner and the rest 4 were married couples. Premarital couples were significantly discordant than married couples, AOR = 1.68; 95%CI (1.36- 5.40). HIV discordance was also significantly associated with having number of two or more sexual partners than one sexual partner AOR = 4.06; 95%CI (2.41-10.13).

**Conclusion:**

The study indicated high prevalence of HIV discordance and increased risk of vulnerability. Therefore, couples should be aware of their own and their partners’ sero-status before and after engagement. Again, the following risk reduction methods were recommended: education of discordant couples on 100% correct condom use, and if condom breaks, or if they forget to use, Post-Exposure Prophylaxis must be established; for those who are not volunteer to use condom and/or have a child, early initiation of ART to positive partner.

**Electronic supplementary material:**

The online version of this article (doi:10.1186/1756-0500-7-893) contains supplementary material, which is available to authorized users.

## Background

One of the difficulties with HIV-discordant couples (where one partner is infected and the other is not) is that the increased risk for infection comes from within stable relationship rather than from outside sexual partners. It is important to provide couples with information that being in a monogamous stable relationship does not mean that partners are not at risk for HIV transmission. Prevalence of HIV discordance among married and cohabitating couples in Africa is high, ranging from 3-20% in the general population [1]. For persons in an HIV-discordant relationship, encouraging monogamy without safer sexual behavior will not decrease the risk of HIV transmission. HIV discordance among couples is an increasingly important issue in HIV prevention activities since high proportion of incident HIV infections in sub-Saharan Africa occur within married HIV-discordant couples [2] and few interventions currently target couples [3]. Discordant couples who have received VCT and other interventions have lower sero-conversion rates; however, incidence within these couples remains high, ranging from 3-8% annually. A comprehensive understanding of the experiences of HIV- discordant couples in Africa could inform efforts to improve the efficacy of couples VCT and other interventions for these couples. Transmission within regular, established discordant partnerships is higher than it is within non-regular discordant partnerships [4]. In addition, immunologic research has suggested that cellular immunity and viral characteristics may be associated with HIV-discordance, [5]. HIV discordant couples represent an important target population for HIV prevention, given the high rates of HIV transmission in couples that do not know or disclose their HIV status. HIV discordant couples are also a valuable population for clinical trials evaluating vaccines, microbicides and other HIV prevention interventions. Increased national access to HIV counselling and testing services and health care can facilitate identification of HIV discordant couples for prevention interventions and enrolment into HIV prevention trials, as In view of these public health benefits and the importance of identifying new prevention strategies to reduce HIV transmission in Africa [6].

In Ethiopia, most of the discordant couples reside in urban areas, and the HIV-negative partners are the group at greatest risk of contracting HIV. From 2,674 cohabiting couples were tested for HIV in the 2005 DHS, 1.8% of the total was discordant. In total population, 1% of HIV-negative married men are living with infected wives and 0.8% of HIV-negative married women are living with infected husbands. In urban areas, 5.6% of HIV-negative married men are living with infected wives and 2.2% of HIV-negative married women are living with infected husbands. In rural areas also, 0.6% of HIV-negative married men are living with infected wives and 0.7% of HIV-negative married women are living with infected husbands [7, 8]. A Study conducted in Dessie (Ethiopia) also, in which of all couple types considered, indicated that 9.8% were found to be sero discordant [Wondwossen A, Gail D: *Socio-Demographic and Behavioral Determinants of Sero-Discordance Among Couples Taking HIV Test in Dessie (Ethiopia),* unpublished] and a study conducted at Addis Ababa VCT sites indicated that 6.6% were sero-discordant among the couples [9]. Another studies conducted in Ethiopia also show that out of 276(138 couples) who presented as couple, 6.5% were found to be sero discordant, and significantly higher rate of sero discordance was observed among participants who had more than one life time sexual partner, (AOR = 15.06(2.87-79.17)) [Girma T, Ahmed A: *Assessment of Premarital HIV Testing Results and Discordant Rate and Factors Associated with Them in Public Voluntary Counselling and Testing Centres in Addis Ababa;* unpublished] and 3.6% of sero-discordance was found and among those living together without formal marriage were 15-times more likely to be infected than males (AOR [95% CI] = 15.78[2.38-104.61]) [Gizachew T, Gail D: *Assessment of HIV Sero-Discordance, Sexual Behaviour and Practice of Preventive Behaviour Against HIV Among Premarital Couples Attending VCT in Bahir Dar, Northwest Ethiopia,* unpublished]. Therefore, this study on HIV discordance and its risk factors among couples receiving HIV test in Dilla town, Ethiopia was conducted because the town has significant high potential risk of HIV discordance that may not have different situation and needs immediate attention.

## Methods

### Study design

Institution based cross sectional study primarily with quantitative data was conducted among couples who tested for HIV. Qualitative data was generated from sero discordant couples through in depth interview to elucidate issues not addressed by the quantitative data and to enrich some quantitative findings from February 22, to June 21, 2011.

### Study area

The study was conducted in Dilla town, Ethiopia. Dilla town is the administrative & trading center which is located at a distance of 359 km south of Addis Ababa, the capital of Ethiopia. This area is commonly known for its cash crops like coffee and fruits and food crops [10].

### Settings

This study was conducted in Dilla University referral teaching hospital that is the major public service provider in zone as well as in Dilla town. The hospital serves integrated HIV counseling and testing (HCT) like in VCT unit, Youth friendly service and PMTCT. Dilla town is purposefully selected from 6 weredas (districts) of Gedio administrative zone because it represents both large urban, pre-urban and rural populations, and according to the sixth report of AIDS in Ethiopia, Dilla is the leading town with 9.3% HIV prevalence out of urban and rural ANC sites in south regional state of Ethiopia [11]. All those couples who are living in Dilla town or nearby vicinities who utilize HIV testing service in Dilla hospital was taken as source population. And those couples who have lived together as a partner over at least 1 year period and who have undergone HIV testing in those VCT unit, youth friendly service and PMTCT in ANC clinic within the specified time period was considered as study population. Couples who are older than 15 years of age and choosing to receive their HIV test results together were included. All of the study participants who came for counseling with more than partner were not enrolled. And, those couples and all other clients tested for HIV earlier or later than the specified time period or do not compatible for one or more reasons mentioned in the inclusion criteria. Plasma samples will be tested using the standard testing algorithm (KHB HIV-1/HIV-2; Stat pack HIV-1/HIV-2; and Unigold HIV-1/HIV-2).

#### For quantitative study

The sample size was calculated using the formula for single population proportion, and considering that the prevalence of HIV sero-discordant among couples in Dilla town is to be 9.8% from similar study conducted in Dessie town [Wondwossen A, Gail D: *Socio-Demographic and Behavioral Determinants of Sero-Discordance Among Couples Taking HIV Test in Dessie (Ethiopia),* unpublished], 95% of level of confidence to address majority of sexual partners in the town and 5% of margin of error or degree of accuracy will be tolerated for absolute precision. Therefore, the following sample size formula is used to determine the sample size of study population. Sample size (n) = Z^2^ × P (1-P) / W^2^; Where: Z = level of confidence; P = proportion; and W = margin of error. And, Sample size (n) = (1.96) ^2^ × .0.098 (1–0.098)/(0.05) ^2^ = 134. The study added 15% of calculated sample size to add its power and to compensate for possible non-response rate. Finally, the sample size was 154 couples or 308 individuals. For qualitative study.

Qualitative data was generated from 11 key knowledgeable couples through in depth interview to elucidate issues not addressed by the quantitative data and to enrich some quantitative findings. The sampling unit was the health institution while study units are those couples participated in research. The determined 154 sample size for quantitative study was sampled consecutively from the health institution until the required sample size was attained. To interview the couples, three counselors working as permanent employees of the hospital, 2 supervisors, and 3 data collectors were recruited. They were given orientation as to how to select the study subjects and then fill out the questionnaire (An outline of the quantitative questioners is added as Additional file 1) by the principal investigator before the onset of their activity and after verbal consent is taken. After identifying couples by asking the reason of HIV testing, the interview took place in the counselling room, before the usual session of pre-test counselling. Each member of the couples was interviewed separately with separate questionnaire and data were collected on each participant on one to one basis in a counselling room. Each consenting participant was given a code first; then the test result was registered after post test counselling. The principal investigator and supervisors closely supervised over all data collection duty. To enrich & address issues not uncovered by quantitative data, qualitative data was generated through in-depth interview with purposively selected respondents. The collection of qualitative data was executed just preceded by the quantitative data. Relevant interview guideline was used to facilitate the in-depth interview. The interview was conducted with eligible informants (discordant couples) through counselors, not by directly contacting them for ethical reasons. Totally, 11 in-depth interviews with key knowledgeable respondents (8 female and 3 male) was held each taking average of 20 minutes (An outline of the qualitative questioners is added as Additional files 2, 3, 4, 5 and 6). Pre tested and structured data reviewing format and structured questionnaire compatible to the national standard VCT record format which comprises, socio demographic characteristics, sexual behavior, test result and some other important variables for quantitative data and an open-ended semi-structured in depth interview guide for the qualitative data collection were used. The format, structured questionnaire, and unstructured interview guide line were translated back from English to Amharic (national language of work) to ensure consistency of the meaning. A carefully pre structured questionnaire was translated first into Amharic and back to English to assure its consistency. The questionnaire was pre-tested on VCT clients Dilla health centre in the town, who were not included in the study to assess the clarity of the questions, their sensitiveness as well as understanding of the data collectors and counsellors and very few changes were made. Discussion was held based on the result of the pre-test and accordingly, some amendments were made. Two day training was given to the supervisors and the data collectors on the procedure. The data was checked for completeness, accuracy, clarity, and consistency by the supervisors and the principal investigator on daily basis. Any error or ambiguity and incompleteness were corrected accordingly. The data were intensively cleaned up before analysis. All the collected record sheets were thoroughly scrutinized every day at the end of data collection session by the principal investigator to ensure their compatibility with the inclusion criteria. Ten percent of the data was double entered to check any inconsistencies. The data was cleaned just preceded by entry. The result of those HIV discordant couple was re-tested and was appointed to come and test after 3 months for further confirmation. *The dependent variable was* discordance of HIV sero-status. The i*ndependent variables were: Socio demographic factors, Behavioral factors, Biological factors*: and *Medical factors.*

### Data analysis

After completing a day-to-day data collection, the variables of the quantitative study were pre-coded and entered to the computer using SPSS statistical soft ware version 16.0. Data cleaning was executed with the same software package by running frequency and cross tab. Summary tables, graphs and charts were used for descriptive purpose. Measures of central tendency and dispersion were used to describe various relevant findings. Some of variables will be recoded into different variables for further data analysis. For all statistical significance tests, the cut- off value set was p < 0.05 as this is considered statistically reliable for analysis of such a study. Moreover, odds ratio with 95% confidence interval and multivariate logistic regression analysis was calculated in order to control for possible confounding variables. Multivariate analysis (multiple logistic regressions) was done to know the independent predicators of HIV discordance. Multinomial logistic regression was used to uncover statistically significant associations between explanatory variables and discrete outcome variable. Linear regression and correlation was used for continuous variables of interest. Numbers and proportions were printed out and the accuracy of recording was checked before analysis. All study-specific data was collected and analyses was restricted to couples in one partner was infected with HIV and one partner was HIV negative (discordant). For behavioral and other characteristics associated with couple HIV status, we compared proportions of each characteristic by couple HIV status. In other words, qualitative data was taped and transcribed. Translation, categorization and summarization of data were done subsequently.

### Ethics

This study was approved by Dilla University research and publication office in collaboration with school of health sciences. A carefully pre structured questionnaire was translated first into Amharic and back to English to assure its consistency. The questionnaire was pre-tested on VCT clients, who were not included in the study to assess the clarity of the questions, their sensitiveness as well as understanding of the data collectors and counsellors and very few changes were made. Discussion was held based on the result of the pre-test and accordingly, some amendments were made. Two days training was given to the supervisors and the data collectors on the procedure. The data was checked for completeness, accuracy, clarity, and consistency by the supervisors and the principal investigator on daily basis. Any error or ambiguity and incompleteness were corrected accordingly. The data were intensively cleaned up before analysis. All the collected record sheets were thoroughly scrutinized every day at the end of data collection session by the principal investigator to ensure their compatibility with the inclusion criteria. Ten percent of the data was double entered to check any inconsistencies. The data was cleaned just preceded by entry. The result of those HIV discordant couple was re-tested and then, the discordant couples were counseled to start HIV treatment by HIV counselors. After having their permission, they have been taken to ART center of the hospital for ensuring access to treatment, and they have been put on appropriate regimen of ART treatment. Therefore, the findings of this study would be disseminated to relevant authorities (stake holders), health care workers, and health institutions.

## Result

### Socio demographic characteristics

A total of 154 couples were who met the criteria recruited in the study, however, interviews were conducted with a total of 152 couples since four participants were not volunteer to participate. This gives a response rate of 98.7% in which the final analysis was calculated. All of non-respondents were couples and concordant negative for HIV infection. One hundred two (33.6%), 104(34.2%) and 98(32.2%) of the study participants were from VCT unit, ANC clinic and youth friendly service, respectively. All of participants presented as couple. The mean age for male and female respondents was 24.4 + 5.2 SD & 21.8 + 4.3 SD with a median age of 25 and 20 years respectively. About 41.1% of the respondents fell within the age range of 21–25 years & 24.3% were aged 15–20 years. Among the total participants, 152(50.0%) were females. The majority 143(47%) of the participants were protestant followed by Orthodox 103(33.9%) and Muslim 42(13.8%) by religion. With regard to ethnicity, 113(37.2%) were Gedio followed by Amhara 74(24.3%), and Guragie ethnic group 57(18.8%). Ninety eight (32.2%) and 88 (28.3%) of the respondents had educational level of primary [1–8] education and tertiary (College/University) education respectively and 23(7.6%) were illiterate. One hundred eighty three (60.2%) of the study participants were employed and the rest were unemployed. Two hundred twenty four (73.7%) of the participants were from Urban and the rest were from rural area and the largest proportion of couples 142 (46.7%) was married, followed by pre sexual 124 (40.8%) (Table 1).

**Table 1:** **Socio demographic characteristics of couples receiving HIV test in Dilla town, Ethiopia, 2011**

Characteristics	Male (n = 152)	Female (n = 152)	Total (=304)
Age group			
15-20	11( 7.2%)	63(41.4%)	74(24.3%)
21-25	68(44.7%)	57(37.5%)	125(41.1%)
26-30	27(17.8%)	22(14.5%)	49(16.1%)
31-35	22(14.5%)	7( 4.6%)	29( 9.5%)
36-40	17(11.2%)	1( 0.7%)	18( 5.9%)
41-45	1( 0.7%)	0( 0.0%)	1( 0.3%)
46-50	4( 2.6%)	2( 1.3%)	6( 2.0%)
>50	2( 1.3%)	0( 0.0%)	2( 0.7%)
Couple type			
Married	71(46.7%)	71(46.7%)	142(46.7%)
Premarital	11( 7.2%)	11( 7.2%)	22( 7.2%)
Pre sexual	62(40.8%)	62(40.8%)	124(40.8%)
Sex partner	7( 4.6%)	7( 4.6%)	14( 4.6%)
Others	1( 0.7%)	1( 0.7%)	2( 0.7%)
Residence			
Urban	112(73.7%)	112(73.7%)	224(73.7%)
Rural	40(26.3%)	40(26.3%)	80(26.3%)
Educational status			
Illiterate	9( 5.9%)	14(9.2%)	23(7.6%)
Able to read	6( 3.9%)	4(2.6%)	10(3.3%)
Primary [1–8]	44(28.9%)	54(35.5%)	98(32.2%)
Secondary (9–110)	23(15.1%)	39(25.7%)	62(20.4%)
Preparatory [11, 12]	17(11.2%)	8(5.3%)	25(8.2%)
Tertiary (College/University)	53(34.9%)	33(21.7%)	88(28.3%)
Employment status			
Employed	119(78.3%)	64(42.1%)	183(60.2%)
Unemployed	33(21.7%)	88(57.9%)	121(39.8%)
Religion			
Orthodox	55(36.2%)	48(31.6%)	103(33.9%)
Catholic	4(2.6%)	12(7.9%)	16(5.3%)
Muslim	20(13.2%)	22(14.5%)	42(13.8%)
Protestant	73(48.0%)	70(46.1%)	143(47.0%)
Ethnicity			
Gedio	55(36.2%)	58(38.2%)	113(37.2%)
Amara	43(28.3%)	31(20.4%)	74(24.3%)
Sidama	11(7.2%)	16(10.5%)	27(8.9%)
Guragie	27(17.8%)	30(19.7%)	57(18.8%)
Others	16(10.5%)	17(11.2%)	33(10.9%)

### HIV sero-prevalence and sero-discordance

HIV sero-prevalence in this study was found to be 11(3.6%). The prevalence in females 8(5.3%) was higher than that in males 3(2.0%). Out of all couples 9(5.9%) were found to be sero-discordant, 2(1.3%) concordant positive and 141(92.8%) concordant negative. Among the different age groups, the highest prevalence 4(36.4%) of HIV infection was observed in age group of 21–25 years and 4(44.4%) of discordant couples were observed in the age group of 21–25. Of the 9 sero-discordant couples, 7(4.6%) were females and 2(1.3%) were males. And, among 9 discordant couples, the highest HIV infection 5(55.6%) was observed among premarital and 4(44.4%) was observed among married couples (Table 2).

**Table 2:** **Prevalence of sero-status and sero-discordance across socio-demographic variables in Dilla town, Ethiopia, 2011**

Variables	Couple HIV sero prevalence						Couple HIV status (152 males & 152 females) = 152 couples				
	Female (n = 152)		Male (n = 152)		Total (n = 304)		Concordant & discordant negative		Discordant positive		
	Positive (n = 8)	Negative (n = 144)	Positive (n = 3)	Negative (n = 149)	Positive (n = 11)	Negative (n = 293)	Female (n = 145)	Male (n = 150)	Female (n = 7)	Male (n = 2)	Total (n = 9)
Age group (yrs)											
15-20	0(0.0%)	11(7.2%)	1(0.7%)	62(40.8%)	1(0.3%)	73(24.0%)	11(7.2%)	62(40.8%)	0(0.0%)	1(0.7%)	1(0.7%)
21-25	4(2.6%)	64(42.1%)	0(0.0%)	57(37.5%)	4(1.3%)	121(39.8%)	64(42.1%)	57(37.5%)	4(2.6%)	0(0.0%)	4(2.6%)
26-30	1(0.7%)	26(17.1%)	2(1.3%)	20(13.2%)	3(1.0%)	46(15.1%)	26(17.1%)	21(13.9%)	1(0.7%)	1(0.7%)	2(1.3%)
31-35	1(0.7%)	21(13.8%)	0(0.0%)	7(4.6%)	1(0.3%)	28(9.2%)	22(14.5%)	7(4.6%)	0(0.0%)	0(0.0%)	0(0.0%)
36-40	0(0.0%)	17(11.2%)	0(0.0%)	1(0.7%)	0(0.0%(	18(5.9%)	17(11.2%)	1(0.7%)	0(0.0%)	0(0.0%)	0(0.0%)
41-45	0(0.0%)	1(0.7%)	0(0.0%)	0(0.0%)	0(0.0%)	1(0.3%)	1(0.7%)	0(0.0%)	0(0.0%)	0(0.0%)	0(0.0%)
46-50	2(1.3%)	2(1.3%)	0(0.0%)	2(1.3%)	2(0.7%)	4(1.3%)	2 (1.3%)	2(1.3%)	2(1.3%)	0(0.0%)	2(1.3%)
>50	0(0.0%)	2(1.3%)	0(0.0%)	0(0.0%)	0(0.0%)	2(0.7%)	2 (1.3%)	0(0.0%)	0(0.0%)	0(0.0%)	0(0.0%)
Couple type											
Married	3(2.0%)	66(43.4%)	2(1.3%)	71(46.7%)	5(1.6%)	137(45.1%)	67(44.1%)	71(46.7%)	2(1.3%)	2(1.3%)	4(2.6%)
Premarital	5(3.3%)	8(5.3%)	0(0.0%)	9(5.9%)	5(1.6%)	17(5.6%)	8(5.3%)	9(5.9%)	5(3.3%)	0(0.0%)	5(3.3%)
Pre sexual	0(0.0%)	63(41.4%)	1(0.7%)	60(39.5%)	1(0.3%)	123(40.5%)	63(41.4%)	61(40.2%)	0(0.0%)	0(0.0%)	0(0.0%)
Sex partner	0(0.0%)	6(3.9%)	0(0.0%)	8(5.3%)	0(0.0%)	14(4.6%)	6(3.9%)	8(5.3%)	0(0.0%)	0(0.0%)	0(0.0%)
Others	0(0.0%)	1(0.7%)	0(0.0%)	1(0.7%)	0(0.0%)	2(0.7%)	1(0.7%)	1(0.7%)	0(0.0%)	0(0.0%)	0(0.0%)
Residence											
Urban	6(3.9%)	99(65.1%)	3(2.0%)	117(77.0%)	9(3.0%)	216(71.1%)	100(65.8%)	118(77.7%)	5(3.3%)	2(1.3%)	7(4.6%)
Rural	2(1.3%)	45(29.6%)	0(0.0%)	32(21.1%)	2(0.7%)	77(25.3%)	45(29.6%)	32(21.1%)	2(1.3%)	0(0.0%)	2(1.3%)
Educational status											
Illiterate	1(0.7%)	8(5.3%)	1(0.7%)	13(8.6%)	2(0.7%)	21(6.9%)	9(6.0%)	13(8.6%)	0(0.0%)	1(0.7%)	1(0.7%)
Able to read	0(0.0%)	6(3.9%)	0(0.0%)	4(2.6%)	0(0.0%)	10(3.3%)	6(3.9%)	4(2.6%)	0(0.0%)	0(0.0%)	0(0.0%)
Primary [1–8]	3(2.0%)	41(27.0%)	0(0.0%)	54(35.5%)	3(1.0%)	95(31.3%)	41(27.0%)	54(35.5%)	3(2.0%)	0(0.0%)	3(2.0%)
Secondary (9–110)	2(1.3%)	21(13.8%)	1(0.7%)	38(25.0%)	3(1.0%)	59(19.4%)	21(13.8%)	38(25.0%)	2(1.3%)	1(0.7%)	3(2.0%)
Preparatory [11, 12]	1(0.7%)	16(10.5%)	0(0.0%)	8(5.3%)	1(0.3%)	24(7.9%)	16(10.5%)	8(5.3%)	1(0.7%)	0(0.0%)	1(0.7%)
Tertiary (College/Univer.)	1(0.7%)	52(34.2%)	1(0.7%)	32(21.1%)	2(0.7%)	84(27.6%)	52(34.2%)	33(21.8%)	1(0.7%)	0(0.0%)	1(0.7%)
Employments status											
Employed	6(3.9%)	113(74.3%)	2(1.3%)	62(40.8%)	8(2.6%)	175(57.6%)	114(75.0%)	63(41.5%)	5(3.3%)	1(0.7%)	6(4.0%)
Unemployed	2(1.3%)	31(20.4%)	1(0.7%)	87(57.2%)	3(1.0%)	118(38.8%)	31(20.4%)	87(57.2%)	2(1.3%)	1(0.7%)	3(2.0%)
Religion											
Orthodox	3(2.0%)	52(34.2%)	2(1.3%)	46(30.3%)	5(1.7%)	98(32.2%)	52(34.2%)	46(30.3%)	3(2.0%)	2(1.3%)	5(3.3%)
Catholic	0(0.0%)	4(2.6%)	0(0.0%)	12(7.9%)	0(0.0%)	16(5.3%)	4(2.6%)	12(7.9%)	0(0.0%)	0(0.0%)	0(0.0%)
Muslim	0(0.0%)	20(13.2%)	0(0.0%)	22(14.5%)	0(0.0%)	42(13.8%)	20(13.2%)	22(14.5%)	0(0.0%)	0(0.0%)	0(0.0%)
Protestant	5(3.3%)	68(44.7%)	1(0.7%)	69(45.4%)	6(2.0%)	137(45.1%)	69(45.4%)	70(46.1%)	4(2.6%)	0(0.0%)	4(2.6%)
Ethnicity											
Gedio	5(3.3%)	50(32.9%)	0(0.0%)	58(38.2%)	5(1.7%)	108(35.5%)	51(33.6%)	58(38.2%)	4(2.6%)	0(0.0%)	4(2.6%)
Amara	3(2.0%)	40(26.3%)	1(0.7%)	30(19.7%)	4(1.3%)	70(23.0%)	40(26.3%)	30(19.7%)	3(2.0%)	1(0.7%)	4(2.6%)
Sidama	0(0.0%)	11(7.2%)	2(1.3%)	14(9.2%)	2(0.7%)	25(8.2%)	11(7.2%)	15(9.9%)	0(0.0%)	1(0.7%)	1(0.7%)
Guragie	0(0.0%)	27(17.8%)	0(0.0%)	30(19.7%)	0(0.0%)	57(18.8%)	27(17.8%)	30(19.7%)	0(0.0%)	0(0.0%)	0(0.0%)
Others	0(0.0%)	16(10.5%)	0(0.0%)	17(11.2%)	0(0.0%)	33(10.9%)	16(10.5%)	17(11.2%)	0(0.0%)	0(0.0%)	0(0.0%)
Total	8(5.3%)	144(94.7%)	3(2.0%)	149(98.0%)	11(3.7%)	293(96.3%)	145(95.4%)	150(98.7%)	7(4.6%)	2(1.3%)	9(5.9%)

### Socio-demographic characteristics and HIV sero-discordance

The prevalence of sero discordant among employed was 6(66.7%) [Crude odds ratio COR = 1.50; 95%CI (1.28 to 2.00)] while among unemployed was 3(33.3%). Among study participants with educational status of primary and less were more likely to be sero discordant, 4(44.4%) [COR = 1.10; 95%CI (0.35-0.46)] than who were secondary, preparatory and above. Another association that was seen among females was prevalence of HIV discordance 7(77.8%) with [(COR = 1.27 95%CI (0.83 to 0.93)]. Based on the residence, those who are living in urban areas 7(77.8%) more likely discordant than rural areas with COR = 3.79; 95% (1.46 to 2.37). After multivariate analysis the only association was seen among premarital couples that was significantly discordant than married couples, AOR = 1.20; 95%CI (1.36- 5.40) (Table 3).

**Table 3:** **Association of selected socio-demographic characteristic and HIV discordance among couples visiting VCT centres in Dilla town, Ethiopia, 2011(n = 304)**

Variables	Total study couples No. (%)	HIV discordance No. (%)	Crude OR (95%CI)	Adjusted OR (95%CI)
Age (years)				
<20	74(24.3)	1(11.1)	1	
20-30	174 (57.2)	6(66.7)	0.19(0.12 to1.69)	
31-40	47 (15.5)	0( 0.0)	0.40(0.14 to1.67)	
> 40	9 (3.0)	2(22.2)	0.63(0.34 to2.03)	
Total	304(100.0)	9(100.0)		
Sex				
Male	152 (50.0)	2(22.2)	1	
Female	152 (50.0)	7(77.8)	1.27(0.83 to0.93)	
Total	304(100.0)	9(100.0)		
Marital status				
Currently not in marital union	162 (53.3)	5 (55.6)	1.11(1.21 to5.32)	1.20(1.36 to 5.40)
Currently in marital union	142 (46.7)	4 (44.4)	1	1
Total	304(100.0)	9(100.0)		
Religion				
Orthodox	103 (33.9)	5 (55.6)	0.81(0.25 to2.65)	
Muslim	42 (13.8)	0 (0.0)	0.48(0.12 to1.93)	
Protestant	143 (47.0)	4 (44.4)	0.61(0.19 to2.00)	
Catholic	12 4.0)	0 ( 0.0)	1	
Total	304(100.0)	9 (100.0)		
Ethnicity				
Gedio	113 (37.2)	4 (44.4)	1.34(1.03 to9.84)	
Amhara	74 (24.3)	4 (44.4)	1.77(0.62 to5.04)	
Sidama	27 (8.9)	1 (11.1)	1.07(0.34 to3.36)	
Guragie	57 (18.8)	0 (0.0)	1.14(0.38 to3.45)	
Others	33 (10.9)	0 ( 0.0)	1	
Total	304(100.0)	9(100.0)		
Educational status				
Primary & less	131 (43.1)	4 (44.4)	1.10(0.35 to0.46)	
Secondary	62 (70.8)	3 (33.3)	0.95(0.45 to1.98)	
Preparatory & above	111 (7.1)	2 (22.2)	1	
Total	304(100.0)	9(100.0)		
Residence				
Urban	225(74.0)	7 (77.8)	3.79(1.46 to2.37)	
Rural	79(26.0)	2 (22.2)	1	
Total	304(100.0)	9(100.0)		
Employment status				
Employed	183(60.2)	6 (66.7)	1.50(1.28 to2.00)	
Unemployed	121(39.8)	3 (33.3)	1	
Total	304(100.0)	9(100.0)		

### Experiences and knowledge on HIV and sexually transmitted Infections

A total of 257 (84.5%) respondents had adequate knowledge on mode of HIV transmission. Two hundred eighty eight (94.7%) of the respondents correctly mentioned at least two mode of HIV transmission. Two hundred eighty five (93.8%) of the respondents correctly mentioned at least two means of HIV prevention. Two hundred sixty four (86.8%) and 270(88.8%) of the study subjects mentioned that abstinence and staying with one partner as a means of HIV prevention method respectively. About 186(61.2%) respondents also mentioned use of condom as means of HIV prevention method. Two hundred thirty three (76.7) of respondents reported having heard about other STIs other than HIV. Only 9(3.0%) of the respondents reported having STIs in the past one year. Almost all 301(98.9%) of them received their result of the test.

### Condom use and sexually transmitted Infection

To assess the extent of condom use from the beginning of sexual exposure, respondents were asked whether they had used condoms the first time they had sex. Only 8 (2.6%) used condoms during their first sexual encounter: 5(3.3%) males and 3(2.0%) females. On the other hand the couples who do not use condom at the last sex with their sexual partner are more likely associated with HIV discordance 7(77.8%) with COR = 1.18; 95% CI (1.71 to 2.44). The main reason not to use condom among non-condom users was trust the partner and 182(59.9%) of the respondents never discussed with their sexual partner about the number of children they to have (Table 4).

**Table 4:** **Risk factors associated with HIV discordance among couples visiting VCT centers in Dilla town, 2011 (n = 152 couples or 304 participants)**

Variables	Total study couples No. (%)	HIV discordance No. (%)	Crude OR (95% CI)	Adjusted OR (95% CI)
Living together				
Yes	184 (60.5)	5(55.6)	3.89(1.25 to 8.38)	
No	120 (39.5)	4(33.1)	1	
Total	304(100.0)	9(44.4)		
Had sex with someone else in past 12 months				
Yes	99 (32.6)	6(66.7)	1.03(0.75 to 1.71)	
No	205 (67.4)	3(33.3)	1	
Total	304(100.0)	9(100.0)		
Need for more children				
Yes	103 (33.9)	6(66.6)	2.66(0.56 to 8.29)	
No	201 (66.1)	3(33.3)	1	
Total	304(100.0)	9(100.0)		
Condom use at last sex act with sexual partner				
Yes	214 (70.4)	2(22.2)	1	
No	90 (29.6)	7(77.8)	1.18(1.71 to 2.44)	
Total	304(100.0)	9(100.0)		
Reported STD in the last 6 month				
Yes	13 (4.3)	2 (22.2)	0.74(0.49 to 1.14)	
No	291 ( 95.7)	7 (77.8)	1	
Total	304(100.0)	9(100.0)		
Ever received blood transfusion				
Yes	16 (5.3)	1 (11.1)	0.58(0.36 to 1.92)	
No	288 (94.7)	8 (88.9)	1	
Total	304(100.0)	9(100.0)		
Desires children				
S/he doesn’t want	276 (90.8)	4 (44.4)	1	
S/he definitely wants	28 (9.2)	5 (55.6)	1.08(0.59 to 1.97)	
Total	304(100.0)	9(100.0)		
Current number of sexual partner				
One or less	255 (83.9)	3 (33.3)	1	1
Two or less	49 (16.1)	6 (66.7)	3.89(2.03 -10.01)	4.06(2.41 to 10.13)
Total	304(100.0)	9(100.0)		
Discussion with partner about Number of children to have				
Yes	122 (40.1)	2(22.2)	1.10(0.65 to 1.58)	
No	182 (59.9)	7 (77.8)	1	
Total	304(100.0)	9(100.0)		
Had children after knowing status				
Yes	34 (11.2)	2(22.2)	1.56(0.50 to 3.14)	
No	270 (88.8)	7(77.8)	1	
Total	304(100.0)	9(100.0)		
Duration of union				
</= 3 years	54 (17.8)	5(55.6)	0.66(0.10 to 1.54)	
4- 9 years	155 51.0)	2 (22.2)	0.42(0.42 to 1.91)	
>9 years	98 (31.2)	2 (22.2)	1	
Total	304(100.0)	9(100.0)		

### HIV discordance and factors associated with HIV discordance

To assess factors associated with HIV discordance, respondents with discordant test result were compared for different variables. In this study it was found that those couples that were living together were at a higher risk of acquiring HIV infection than those not living together with COR =3.89; 95% CI (1.25-8.38). To investigate the impact of other variables on outcome variable multivariate logistic regression was used. In this analysis the only factor that is significantly associated with HIV discordance is having number of two or more sexual partners than one sexual partner AOR = 4.06; 95%CI (2.41-10.13) (Table 4).

### Result (qualitative)

1. Explanations for couple discordance

Very few clients stated accurate information about why HIV- discordance exists. Some clients explained that HIV transmission was based on luck, but their luck could end at one time. Majority of discordant couples shocked by their HIV- discordant results and they were unsure of the explanation. In addition, mixtures of correct and incorrect explanations were common. One of the responses for the reason why discordance exists, the common initial response was that they had no explanation. In some cases, denying the existence of HIV-discordance. *“…It is extremely difficult to understand how discordance can exist and still I have failed to believe that I am HIV- positive and my partner is negative, it is too hard since we had unprotected sex a lot of times for years…”* HIV-positive woman.

2. Sexual practices and intimacy

After receiving HIV discordant results, couples experienced blame each other about bringing HIV into their life. As one HIV-positive female reported blame from her partner: *“My husband says this is my fault since we had no HIV test before our marriage. I am sure that you were positive before our marriage. As you know me I am decent person, and thanks to God I refuse to have sex with you for the future…”*

On other hand, most discordant couples viewed prevention of HIV transmission as the most important thing. They suggested using condom as the best alternative available for protecting the negative partner. *“…we should use condom because we have to live together and protect my partner. So far no change in terms of love-making…”* HIV-positive female

*“We will use condoms all the time and I want to see and check that he has put it correctly before we go to bed….”* HIV-negative female

However, some respondents said that there is a tendency to increase sexual intercourse after discordant result is known.*“…The frequency of sex has been increased above the usual. Having safe sex is normal and helps for bonding…”* HIV-positive male

3. Coping strategies and prevention plan by discordant couples

The two broader themes emerged from the in-depth interviews and the qualitative analyses were: sexual practices for HIV prevention and how to maintain the relationship. Two types of sexual practices emerged under the first theme: 1) abstinence, and 2) consistent condom use. Discordant couples (especially those who are HIV positive) are emotionally disturbed just after disclosure of test result. Therefore, they hardly accept coping strategies and HIV infection prevention plan. One of HIV negative male respondent said that: ***“…****Whenever two partners have discordant result; it just protection from God or what matters is that there may be individuals who have different gene that is not attacked by HIV virus…”*

Despite questioning their discordance, most couples had plan and developed prevention strategies. One HIV-positive male couple said: ***“…****This is just a gift from God. You can do nothing. The only thing that I can do is that if my wife agrees, I want to live with her. In our next life, safe sex by using condom is the only option. Because she has to remain for our child after I have passed…”*

Two reasons for securing the relationship despite HIV sero-discordance also emerged: 1) for the life of their children that they have previously and 2) availability of condom. One discordant negative father said that:*” I love our children and I still need her help to support and be with our children and I want to live with her, but I am scared to be infected… that’s the only challenge that I have…”*

4. Disclosure of HIV discordant test result

8(88.9%) of participants had disclosed their HIV status to their partner. The use of disclosure to sexual partner was explained by one of HIV positive man: *“…I am happy talking to my partner, because I know she loves me. She is actually safe because she knew my status and I knew hers, so we have to protect ourselves…”*

## Discussion

The prevalence of sero-discordance was found to be 5.9% which is higher than national EDHS 2005 survey (1.8%) among cohabiting couples [8]. However, this was lower than a study in Dessie (9.8%) among couples [Wondwossen A, Gail D: *Socio-Demographic and Behavioral Determinants of Sero-Discordance Among Couples Taking HIV Test in Dessie (Ethiopia),* unpublished], and urban residents in EDHS 2005 survey (10.9%) among cohabiting couples [8]. This might be explained by differences in study population. Prevalence of sero-discordance among never married (premarital) couples is still lower 5/152(3.3%); AOR = 0.83; 95%CI (0.47 to 1.46) as compared to a study in Dessie among never married couples (most premarital) (6.2%) (95% CI = [0.05-0.08]) [Wondwossen A, Gail D: *Socio-Demographic and Behavioral Determinants of Sero-Discordance Among Couples Taking HIV Test in Dessie (Ethiopia),* unpublished]. When compared to African countries it is also lower i.e. 15% among Kenyan premarital couples [12] and 8-31% in Eastern and southern African countries [6]. This might be attributed to temporal and spatial differences between study populations.

The prevalence of sero-positive women among discordant was found to be 77.8% which is higher than a similar study done in Dessie (50%) and Kampala (59%) at VCT centres [Wondwossen A, Gail D: *Socio-Demographic and Behavioral Determinants of Sero-Discordance Among Couples Taking HIV Test in Dessie (Ethiopia),* unpublished, [13]. Female partners among discordant couples had a median age of 22.4 and a range of 23 years, while women study subjects in a study in Dessie had a median age of 20 and a range of 32 years. Male partners among discordant couples had median age of 22, while it was 28 in Dessie [Wondwossen A, Gail D: *Socio-Demographic and Behavioral Determinants of Sero-Discordance Among Couples Taking HIV Test in Dessie (Ethiopia),* unpublished]. Educational status was not found to be significantly associated with sero-status of study subjects, which is similar to a study in Dessie and in Kampala [Wondwossen A, Gail D: *Socio-Demographic and Behavioral Determinants of Sero-Discordance Among Couples Taking HIV Test in Dessie (Ethiopia),* unpublished,13]. Similarly in this study occupational status and age category had no significantly associated with sero-status of study participants unlike a study in Dessie. This study showed that sero discordance was significantly associated with premarital sex and number of lifetime sexual partner. This is also shown to be a risk factor for HIV sero positivity. This may lead to consideration of the prevalence of sero discordance among premarital couples and suggests the need for further investigations. Higher rate of sero discordance was observed among study participants who had history of premarital sex 5(55.6%) than those without history marriage 4(44.4%). This is in line with the finding of another study [Wondwossen A, Gail D: *Socio-Demographic and Behavioral Determinants of Sero-Discordance Among Couples Taking HIV Test in Dessie (Ethiopia),* unpublished] in which 13.7% of married couples were found to be discordant, while the prevalence of sero discordance among never married were 53(6.2%) This result signifies the importance of HIV testing prior to marriage particularly when couples intend for marital union.

VCT for couples, including premarital couples, and married couples, is a critical component of prevention activities. Once identified, HIV-discordant can take precautionary measures to prevent transmission to their partners or to prevent acquisition. To date, HIV prevention messages in Ethiopia have reinforced the idea that abstinence, faithfulness, and using condom provide safety from HIV infection. Our finding that living together is not guarantee for not being infected by HIV within couples that underscores the importance of adding a strong message promoting couples testing as part of the “being faithful” message. This indicates a need of more complex prevention messages if Ethiopia is to be able to further reduce the incidence of HIV infection in the context couple HIV prevention strategy. To help control the HIV epidemic in Ethiopia, HIV-discordant couples identified in VCT programs should be provided with basic preventive care, antiretroviral therapy, and specially designed counselling interventions for couples.

### Limitation of the study

Due the rare nature of the study, getting large number couples coming for VCT was difficult. But we waited to collect data for 4 months. Therefore, small sample size of the study was one of limitation of this study. In other words, exclusion of persons with multiple sexual partners was done since it was difficult to consider someone as a couple if he or she had multiple sexual partners, but it had influence in getting sufficient number of couples.

## Conclusion

This study indicated that significant prevalence of HIV discordance among couples and showed an increased risk of vulnerability to HIV infection. Prevalence of sero discordant was significantly associated with premarital sexual couples and number of lifetime sexual partner. Discordance was high and discordant couples were 4 times more common than concordant positive couples.

Discordance rates were also highest within married couples. 9/11(81.8%) of HIV-infected couple members had an HIV negative partner. Challenges for discordant couples observed include: reproductive desire, fear of separation, and sexual activity or practices. As coping strategies often used by couples include: condom use, abstinence, on-going counseling, and reducing frequency of sex.

The findings of this study are supported by evidences that suggest that a woman’s greatest risk of contracting HIV lies within a marital relationship. Fewer attempts have been made to understand a man’s risk within marriage; but, in regions with generalised epidemics, high rates of sero-discordance among heterosexual couples in which the woman is HIV-positive suggest that a man’s risk of marital HIV acquisition could also be substantial. Premarital couples in general and discordant couples in particular should be aware of the presence of possible risk reduction strategies. Therefore, based on the conclusion, the following recommendations are forwarded

1. Risk reduction methods among discordant couples

- Providing support to HIV-infected partner in every aspect,
- Providing psychological support for one may feel guilt for bringing HIV into the relationship, possibility of blame, abandonment, especially if woman is infected.
- Education of discordant couples on 100% correct condom use during sexual intercourse,
- If condom breaks, or if they forget to use one, talk to their counsellor about PEP, “Post-Exposure Prophylaxis” must be established;
- For those discordant couples who are not volunteer to use condom, early initiation of ART can make it very unlikely that positive partner will pass HIV infection to negative partner (Counsel and early initiation of ART for discordant couple)
- For a woman who is HIV positive and wants to have a child from HIV negative partner, the system for artificial insemination, placing the man’s sperm into the woman’s vagina allows pregnancy without exposing the man to HIV is important
- Intervention programs specifically targeting discordant couples should be designed to provide accurate and timely information on ‘HIV discordance’ at various VCT entry points;
- Even ccounseling messages for VCT Couples should include: for discordant couples: that is on safe sex (condom use and mutual masturbation), and abstinence.

2. Risk reduction methods among general public

- Couple HIV testing should be promoted for in VCT sites;
- Every effort should be exerted to make couples or premarital couples aware of their own and their partners’ sero status before engagement or marriage;
- HIV testing is highly recommended for couples who intend for marriage and even within marriage;

3. Overall recommendations

- Study needs to be conducted to further investigate prevalence and determinants of the infection and discordant rate among premarital clients,
- As the current study included only VCT clients from public VCT centre; further research should be carried on discordant couples to understand coping skills, reproductive choices and rights violations; to insight on challenges and issues confronting discordant couples.

## Electronic supplementary material

Additional file 1: **Outline of general information questioner.** (DOC 126 KB)

Additional file 2: **General in-depth interview questioner.** (DOC 73 KB)

Additional file 3: **In-depth interview questioner for exploring the paradox from discordant couples.** (DOC 114 KB)

Additional file 4: **In-depth interview questioner for exploring the paradox from counsellors.** (DOC 102 KB)

Additional file 5: **FGD questioner for exploring the paradox from discordant couples.** (DOC 92 KB)

Additional file 6: **FGD questioner for exploring the paradox from counsellors.** (DOC 88 KB)
