# Supplementary material for: Assessment of HIV discordance and associated risk factors among couples receiving HIV test in Dilla, Ethiopia
Source: BMC Res Notes. 2014 Dec 10;7:893. doi: 10.1186/1756-0500-7-893 (PMC4295257; doi:10.1186/1756-0500-7-893)
Supplement: Supplementary file 1 — Additional file 1: Outline of general information questioner. (DOC 126 KB) [file 13104_2012_3387_MOESM1_ESM.doc]

Tool 01

Questionnaire No: _______

**QUESTIONNAIRE FOR HIV DISCORDANCE AND ASSOCIATED FACTORS**

**AMONG COUPLES IN DILLA TOWN**

Couple code____________________

Code of Respondent: ...................................................................

Name of Interviewer/ Counselor: ...................................................................

Code of VCT centre/health facility/Location of Interview: ...................................................................

Date of Interview: ...................................................................

Time of Interview: ...................................................................

Instructions: *Please answer all the questions below to the best of your knowledge. Where boxes are provided tick* [√] *the most appropriate one.*

**How to fill the questionnaire**

1. This questionnaire has 6 parts.
2. Each question has its own serial number, question, and answer
3. Part 1. 11 questions
4. Part 2. 22 questions
5. Part 3. 4 questions
6. Part 4. 6 questions
7. Part 5. 3 questions and
8. Part 6. 7 questions.
9. Generally they are 53 questions.
10. After reading the question with its possible answer provided, Please try to give response by encircling the choice that fit your answer.
11. After completing the questions, please try to give it back to the person who gave you.

**Site (VCT center) type**

1. Integrated ❐
2. Free standing ❐
3. NGO ❐
4. Youth ❐
5. Mobile ❐
6. Private ❐
7. Home-based ❐
8. Work place ❐
9. Governmental ❐
10. Other (specify) ______________________________

Tool 01

**QUESTIONNAIRE TO HIV DISCORDANT COUPLES**

**CONSENT FORM**

Hello my name is _______________________ and I work for an organization named Dilla university school of health sciences found in Dilla town. I am here to collect information for the research to be conducted on HIV discordance and associated factors. The purpose of the study is to understand the associated factors with occurrence of HIV discordance among sexual partners and establish evidence and support the activities carried out to posive prevention strategies in Dilla town as well as in the country.The questionnaire will take 20-30 minutes.

In the questionnaire you’ll be asked some very personal questions that some people find it difficult to answer. Your name will not be written on this questionnaire, and will never be used in connection with any of the information you tell me. You are selected for this survey merely by chance, not done intentionally.

Participation is based on your willingness besides; you can withdraw from the study anytime. However your kin participation would be very useful. In addition, no personal identification will be written and we assure you that what ever information you are providing will only be used for the research purpose and the data will be handled only by the research team. While we are collecting the data it is difficult to jot down everything thus we will tape record our discussion.

**Participant’s statement**

I know what this research study is about and I know what will do if choose to take part. I have had a chance to ask question and I know I can ask question at any time during or after the interview. I know I am free to not answer a question or quit at any time. I freely choose to be a part of this study. If you need any further information about the study please contact the following person.

Moges Tadesse

Dilla University, school of health sciences

Tel: 0911923244

Are you willing to participate in the study?

Agreed __________

Not Agreed ____________

Thank you for your time and contribution.

Name of Data collector ___________________ signature ______________________

Date of data collection _____________

Tool 01

**GENERAL INFORMATION**

Introduction

Thank you for choosing to participate in this research study considering the assessment of HIV discordance and associated factors among sexual partners receiving HIV test in Dilla, Ethiopia. The following questionnaire is designed to collect some basic background information about you will aid in interpretation of the results. Please complete the questions as accurately and honestly as you can. If the question is unclear to you, feel free to ask me what is meant by the question. If there is a question that makes you uncomfortable you can choose not to respond to it or any other question. If you feel an uncomfortable emotional response as a result of the question on this questionnaire, please inform me at once and measures will be taken to reduce your discomfort immediately. Take as much time as you require completing the questionnaire.

Instructions: *Please answer all the questions below to the best of your knowledge. Where boxes are provided tick* [√] *the most appropriate one*

Tool 01

**QUESTIONNAIRE FOR ALL STUDY PARTICIPANTS**

| **PART 1: BACKGROUND CHARACTERSTICS**  **101. How old were you at your last birthday?**  1. _______ Years.  88. Don’t know ❐  99. No response ❐ | **102. What is your sex?**  1. Male ❐  2. Female ❐  99..No response ❐ | **103. What is marital status?**  1. Married ❐  2. Premarital ❐  3. Pre sexual ❐  4. Sex partner ❐  5. Others ______________  99. No response ❐ |
| --- | --- | --- |
| **104. Residence area**  1. Urban ❐  2. Rural ❐  99..No response ❐ | **105. Education Level**  1. Illiterate ❐  2. Able to read ❐  3. Primary (1-8) ❐  4. Secondary (9-10) ❐  5.preparatory (11-12) ❐  6. Tertiary (college/university) ❐  7. Other (specify) ______  99. No response ❐ | **106. Employment status**  1. Employed ❐  2. Unemployed ❐  99. No response ❐ |
| **109. What is your religion?**  1. Orthodox ❐  2. Catholic ❐  3. Muslim ❐  4. Protestant ❐  5. No religion ❐  6. Other (specify)_________  99. No response ❐ | **108. What is your ethnicity?**  1. Gedio ❐  2. Amhara ❐  3. Sidama ❐  4. Guragie ❐  5. Other (specify)__________  99. No response ❐ | **107. Do you have already children?**  1. Yes ❐ Number: _______  2. No ❐  99. No response ❐ |
| **110. Duration in the union?**  1. _______ Years.  88. Don’t know ❐  99. No response ❐ | **111. Number of children with partner**  1. In current relationship-------  2. Previous relationship------------  99. No response ❐ |  |
|  | | |
| **PART 2: SEXUAL HISTORY**  **201Do you need to have children?**  1. S/he doesn’t want ❐  2. S/he wants ❐  99. No response ❐ | **202. Discus with partner about number of children to have?**  1. Yes ❐  2. No ❐  99. No response ❐ | **203. Discus with partner about when to have children?**  1. Yes ❐  2. No ❐  99. No response ❐ |
| **204. History of STI?** 1. Yes ❐  2. No ❐  3. Other_____________  88. Don’t know ❐  99. No response ❐ | **205. Male circumcision status?**  1. Yes ❐  2. No ❐  3. Other_____________  88. Don’t know ❐  99. No response ❐ | **206. Contraception discussed with health provider/counselor**  1. Yes ❐  2. No ❐  99. No response ❐ |
| **207. Pregnancy & HIV discussed with health provider/counselor?**  1.Yes ❐  2. No ❐  99. No response ❐ | **208. Birth spacing and HIV discussed with health provider/counselor?**  1. Yes ❐  2. No ❐  99. No response ❐ | **209. Discussed abortion discussed with health provider/counselor?**  1. Yes ❐  2. No ❐  99. No response ❐ |
| **210. Discussed condom use with health provider or counselor?**  1. Yes ❐  2. No ❐  99. No response ❐ | **211. Currently using contraception?**  1. Yes ❐  2. No ❐  99. No response ❐ | **212. Self/partner on ART?**  1. Yes ❐  2. No ❐  99. No response ❐ |
| **213. Had children after knowing status?**  1. Yes ❐  2. No ❐  99. No response ❐ | **214. Ever had sex after knowing status?**  1. Yes ❐  2. No ❐  99. No response ❐ | **215. Condom use in the last 3 months?**  1. Never ❐  2. Always/consistent ❐  3. Sometimes ❐  4. Other ____________ ❐  99. No response ❐ |
| **216. Is partner sex worker?**  1. Yes ❐  2. No ❐  3. Believes so ❐  88. Don’t know ❐  99. No response ❐ | **217. Used condom in last sex?**  1. Yes ❐  2. No ❐  3. Doesn’t remember ❐  4. Other _________ ❐  99. No response ❐ | **218. Is partner pregnant?**  1. Yes ❐  2. No ❐  88. Don’t know ❐  99. No response ❐ |
| **219. Number of casual partners, if yes, how many?**  1. Yes ❐-------------  2. No ❐  3. Other_____________  88. Don’t know ❐  99. No response ❐ | **220. Number of steady partners, if yes, how many?**  1. Yes ❐------------  2. No ❐  3. Other_________  88. Don’t know ❐  99. No response ❐ | **221. Current number of sexual partner?**  1. One or less ❐  2. Two or more ❐  88. Don’t know ❐  99. No response ❐ |
| **222. Do you use condom during sexual intercourse? How often?**  1. Always ❐  2. Sometimes ❐  3. Rarely ❐  88. Don’t know ❐  99. No response ❐ |  |  |
|  | | |
| **PART 3: MEDICAL HISTORY**  **301. Surgery (minor/major)**  1. Yes ❐  2. No ❐  3. Other_____________  88. Don’t know ❐  99. No response ❐ | **302. Tooth extraction**  1. Yes ❐  2. No ❐  3. Other_____________  88. Don’t know ❐  99. No response ❐ | **303. Blood transfusion** 1. Yes ❐  2. No ❐  3. Other_____________  88 Don’t know ❐  99. No response ❐ |
| **304. Abortion**  1. Yes ❐  2. No ❐  3. Other_____________  88. Don’t know ❐  99. No response ❐ |  |  |
|  |  |  |
| **PART 4: INFORMATION AND ACCESS TO VCT CENTER**  **401. Have ever been tested for HIV before?**  1. Yes ❐  2. No ❐  88. Don’t know ❐  99. No response ❐ | **402. How long ago did you learn your HIV status?**  1. < 6 months ❐  2. 6-12 months ❐  3. 1-2 years ago ❐  4. >2 years ago ❐  88. Don’t know ❐  99. No response ❐ | **403. Rate your own *health* as**  1. Poor ❐  2. Good ❐  3. Very good ❐  4. Excellent ❐  88. Don’t know ❐  99. No response ❐ |
| **404. Hear of the service**  1. Radio ❐  2. Posters ❐  3. Other clients ❐  4. Newspapers ❐  5. Health institution ❐  6. Telephone hotline ❐  7. Anti AIDS clubs ❐  8. Friends & family ❐  9. TV ❐  10. Other_____________  88. Don’t know ❐  99. No response ❐ | **405. Testing history (reason for HIV test) or Primary reason here**  1. Had risk ❐  2. Partner had risk ❐  3. Not trust partner ❐  4. Ill/symptom ❐  5. Premarital ❐  6. Marital reunion ❐  7. Family planning ❐  8. Visa applicant ❐  9. Referred ❐  10. 2nd test ❐  11. Confirm positive result ❐  12. Need counseling ❐  13. Other_____________  88. Don’t know  99. No response ❐ | **406. Client referred by**  1. Self ❐  2. Public health institution ❐  3. Private health institution ❐  4. Military health institution ❐  5. Friend /relative ❐  6. School ❐  7. Religious inst ❐.  8. Client ❐  9. Others ❐  88. Don’t know ❐  99. No response ❐ |
|  | | |
| **PART 5: PRE TEST RESULT EXPECTATION AND PARTNER NOTIFICATION**  **501. Result client expect**  1. Negative ❐  2. Positive ❐  88. Don’t know ❐  99. No response ❐ | **502. Pretest partner notification plan**  1. Refused to notify ❐  2. Agree to notify ❐  3. Plan to notify Unsure ❐  88. Don’t know ❐  99. No response ❐ | **503. HIV test result**  1. Reactive ❐  2. Non reactive ❐ |
| **504. Couple status**  1. Concordant positive ❐  2. Concordant **negative** ❐  3. Discordant ❐ |  |  |
|  | | |
| **PART 6: POST TEST PARTNER NOTIFICATION AND RISK REDUCTION PLAN**  **601. Refused result**  1. Yes ❐  2. No ❐  3. Other_____________  88. Don’t know ❐  99. No response ❐ | **602. Condom accepted**  1. Yes ❐  2. No ❐  3. Not available ❐  88. Don’t know ❐  99. No response ❐ | **603. Risk reduction plan developed**  1.Yes ❐  2. No ❐  3. Other_____________  88. Don’t know ❐  99. No response ❐ |
| **604. Post test partner notification plan**  1. Refused to notify ❐  2. Agree to notify ❐  3. Plan to notify ❐  4. Unsure ❐  5. Other_____________  88. Don’t know ❐  99. No response ❐ | **605. Client referred to**  1. ART ❐  2. Follow up ❐  3. Counselling ❐  4. Social service ❐  5. TB clinic ❐  6. Hospital ❐  7. Family planning ❐  8. Other_____________  88. Don’t know ❐  99. No response ❐ | **606. How long have you been in a sero-discordant relationship?**  1. 1 year ❐  2. 1--3 years ❐  3. 3--5 years ❐  4. 5 years ❐  5. Other_____________  88. Don’t know ❐  99. No response ❐ |
| **607. How often does the HIV uninfected partner in the relationship uptake routine HCT for status confirmation?**  1. Every3months ❐  2. Every 6months ❐  3. Every 12months ❐  5. >12months ❐  6. Other_____________  88. Don’t know ❐  99. No response ❐ |  |  |

**Thank you for taking time to fill in this questionnaire.**

**God richly bless you!**
