# Supplementary material for: Assessment of HIV discordance and associated risk factors among couples receiving HIV test in Dilla, Ethiopia
Source: BMC Res Notes. 2014 Dec 10;7:893. doi: 10.1186/1756-0500-7-893 (PMC4295257; doi:10.1186/1756-0500-7-893)
Supplement: Supplementary file 2 — Additional file 2: General in-depth interview questioner. (DOC 73 KB) [file 13104_2012_3387_MOESM2_ESM.doc]

Tool 02

Schedule No: _____

**AN IN-DEEPTH INTERVIEW GUIDE FOR HIV DISCORDANCE AND ASSOCIATED FACTORS AMONG COUPLES IN DILLA TOWN**

Couple code____________________

Code of Respondent: ...................................................................

Name of Interviewer/ Counselor: ...................................................................

Code of VCT centre/health facility/Location of Interview: ..................................................................

Date of Interview: ...................................................................

Time of Interview: ...................................................................

Instructions: *Please answer all the questions below to the best of your knowledge.*

**How to respond the questionnaire**

1. This questionnaire has 6 parts.
2. Each question has its own serial number, question, and answer
3. Part 1. 6 questions, and
4. Part 2. 4 objectives questions

**Site (VCT center) type**

Integrated ❐

Free standing ❐

NGO ❐

Youth ❐

Mobile ❐

Private ❐

Home-based ❐

Work place ❐

Governmental ❐

Other (specify) ______________________________

Tool 02

**IN-DEPTH INTERVIEW GUIDE FOR HIV DISCORDANCE COUPLES**

**CONSENT FORM**

Hello my name is _______________________ and I work for an organization named Dilla university school of health sciences found in Dilla town. I am here to collect information for the research to be conducted on HIV discordance and associated factors. The purpose of the study is to understand the associated factors with occurrence of HIV discordance among sexual partners and establish evidence and support the activities carried out to posive prevention strategies in Dilla town as well as in the country. The questionnaire will take 20-30 minutes.

In the questionnaire you’ll be asked some very personal questions that some people find it difficult to answer. Your name will not be written on this questionnaire, and will never be used in connection with any of the information you tell me. You are selected for this survey merely by chance, not done intentionally.

Participation is based on your willingness besides; you can withdraw from the study anytime. However your kin participation would be very useful. In addition, no personal identification will be written and we assure you that what ever information you are providing will only be used for the research purpose and the data will be handled only by the research team. While we are collecting the data it is difficult to jot down everything thus we will tape record our discussion.

**Participant’s statement**

I know what this research study is about and I know what will do if choose to take part. I have had a chance to ask question and I know I can ask question at any time during or after the interview. I know I am free to not answer a question or quit at any time. I freely choose to be a part of this study. If you need any further information about the study please contact the following person.

Moges Tadesse

Dilla University, school of health sciences

Tel: 0911923244

Are you willing to participate in the study?

Agreed __________

Not Agreed ____________

Thank you for your time and contribution.

Name of Data collector ___________________ signature ______________________

Date of data collection _____________

**Tool 02**

**PART I: GENERAL INFORMATION**

**DEMOGRAPHIC QUESTIONNAIRE**

Introduction

Thank you for choosing to participate in this research study considering the assessment of hiv discordance and associated factors among sexual partners receiving HIV test in Dilla, Ethiopia. The following questionnaire is designed to collect some basic background information about you will aid in interpretation of the results. Please complete the questions as accurately and honestly as you can. If the question is unclear to you, feel free to ask me what is meant by the question. If there is a question that makes you uncomfortable you can choose not to respond to it or any other question. If you feel an uncomfortable emotional response as a result of the question on this questionnaire, please inform me at once and measures will be taken to reduce your discomfort immediately. Take as much time as you require completing the questionnaire.

Instructions: *Please answer all the questions below to the best of your knowledge. Where boxes are provided tick* [√] *the most appropriate one*

| Participant No | Background information |  |
| --- | --- | --- |
| 101 | Age of participant | 1. _______ Years.  88. Don’t know ❐  99. No response ❐ |
| 102 | Sex | 1. Male ❐  2. Female ❐  3.No response ❐ |
| 103 | Marital status | **What is marital status?**  1. Married ❐  2. Premarital ❐  3. Pre sexual ❐  4. Sex partner ❐  5. Others ______________  6. Nosponse ❐ |
| 104 | Educational status | 1. Illiterate ❐  2. Able to read ❐  3. Primary (1-8) ❐  4. Secondary (9-10) ❐  5.preparatory (11-12) ❐  6. Tertiary (college/university) ❐  7. Other (specify) ______  99. No response ❐ |
| 105 | Religion | 1. Orthodox ❐  2. Catholic ❐  3. Muslim ❐  4. Protestant ❐  5. No religion ❐  6. Other (specify)_________  99. No response ❐ |
| 106 | Occupation | 1. Employed ❐  2. Unemployed ❐  3.No response ❐ |

**Part1 ፡ General information about study participants**

| **Participant** | **Age** | **Sex** | **Marital status** | **Educational status** | **Religion** | **Job status** | **Remark** |
| --- | --- | --- | --- | --- | --- | --- | --- |
| **1** |  |  |  |  |  |  |  |
| **2** |  |  |  |  |  |  |  |
| **3** |  |  |  |  |  |  |  |
| **4** |  |  |  |  |  |  |  |
| **5** |  |  |  |  |  |  |  |
| **6** |  |  |  |  |  |  |  |
| **6** |  |  |  |  |  |  |  |
| **7** |  |  |  |  |  |  |  |
| **8** |  |  |  |  |  |  |  |
| **9** |  |  |  |  |  |  |  |
| **10** |  |  |  |  |  |  |  |
| **11** |  |  |  |  |  |  |  |

Tool 02

**PART II: IN DEPTH INTERVIEW GUIDE**

1. How would you define HIV couple discordance?

2. What is the purpose of couple HIV counselling and testing?

3. Is there couple HIV counselling and testing in your locality? Yes ❐ No❐

| No 201 | **Objective: To explore sexual practices for HIV prevention**  Q1. What type of sexual method do you use for HIV prevention? Why?  **Probe :**   1. How often do you use condom? Why? 2. Have you practice unsafe sex (with out condom)? Why? 3. Have you practiced other alternative non penetrative sexual practices or abstinence? What type? Why? 4. What is there decision regarding future sexual partnership? 5. What types of safe sex do you practic? 6. Do you seek other HIV concordant partners for sexual pleasure? |
| --- | --- |
| 202 | **Objective: To explore desire for Pregnancy with the Dilemma of Condom Use**    **Probe :**   1. Do you need to have child? In what way? Why? 2. Why do you need to be or make pregnant? 3. Influence of the desire to have children on sexual practices |
| 203 | **Objective: To explore the effect of the social net works & health systems to get child**  **Probe :**   1. What do your relatives on yours partner’s relatives say on your HIV status & need of children? 2. What type of advices was given to you to have children from health providers? Have you consulted for this case? |
| 204 | **Objective: To explore the implication of desire of children on HIV transmission of HIV**  **Probe:**   1. What kind of ways do you use to have child? 2. What will happen if you practice unsafe sex to have children? Why? 3. If your partner is not willing to Patrice unsafe sex, what do you do? Why? 4. What recommendations do you have for future efforts such as these? 5. Is there anything more you would like to add? |

**Thank you for taking time to fill in this questionnaire.**

**God richly bless you!**
