# Supplementary material for: Assessment of HIV discordance and associated risk factors among couples receiving HIV test in Dilla, Ethiopia
Source: BMC Res Notes. 2014 Dec 10;7:893. doi: 10.1186/1756-0500-7-893 (PMC4295257; doi:10.1186/1756-0500-7-893)
Supplement: Supplementary file 4 — Additional file 4: In-depth interview questioner for exploring the paradox from counsellors. (DOC 102 KB) [file 13104_2012_3387_MOESM4_ESM.doc]

Tool 04

Schedule No: _____

**AN IN-DEEPTH INTERVIEW GUIDE FOR EXPLORING THE PARADOX: KNOWLEDGE, CHALLENGES, AND COPING CHOICES AMONG HIV COUNSELLORS IN DILLA TOWN**

Code of Interviewer/ Counsellor: ...................................................................

Code of VCT centre/health facility/Location of Interview: ..................................................................

Date of Interview: ...................................................................

Time of Interview: ...................................................................

Instructions: *Please answer all the questions below to the best of your knowledge.*

**How to respond the questionnaire**

1. This questionnaire has 3 parts.
2. Each question has its own serial number, question, and answer
3. Part 1. 6 questions, and
4. Part 2. 18 questions
5. Part 2. 4 objectives questions

**Site (VCT center) type**

Integrated ❐

Free standing ❐

NGO ❐

Youth ❐

Mobile ❐

Private ❐

Home-based ❐

Work place ❐

Governmental ❐

Other (specify) ______________________________

**Tool 04**

**IN-DEPTH INTERVIEW GUIDE FOR EXPLORING THE PARADOX: KNOWLEDGE. CHALLEGES, AND COPING CHOICES AMONG HIV COUNSELLORS**

**CONSENT FORM**

Hello my name is _______________________ and I work for an organization named Dilla university school of health sciences found in Dilla town. I am here to collect information for the research to be conducted on HIV discordance and associated factors. The purpose of the study is to understand the associated factors with occurrence of HIV discordance among sexual partners and establish evidence and support the activities carried out to posive prevention strategies in Dilla town as well as in the country. The questionnaire will take 20-30 minutes.

In the questionnaire you’ll be asked some very personal questions that some people find it difficult to answer. Your name will not be written on this questionnaire, and will never be used in connection with any of the information you tell me. You are selected for this survey merely by chance, not done intentionally.

Participation is based on your willingness besides; you can withdraw from the study anytime. However your kin participation would be very useful. In addition, no personal identification will be written and we assure you that what ever information you are providing will only be used for the research purpose and the data will be handled only by the research team. While we are collecting the data it is difficult to jot down everything thus we will tape record our discussion.

**Participant’s statement**

I know what this research study is about and I know what will do if choose to take part. I have had a chance to ask question and I know I can ask question at any time during or after the interview. I know I am free to not answer a question or quit at any time. I freely choose to be a part of this study. If you need any further information about the study please contact the following person.

Moges Tadesse

Dilla University, school of health sciences

Tel: 0911923244

Are you willing to participate in the study?

Agreed __________

Not Agreed ____________

Thank you for your time and contribution.

Name of Data collector ___________________ signature ______________________

Date of data collection _____________

**Tool 04**

**PART I : GENERAL INFORMATION**

**DEMOGRAPHIC QUESTIONNAIRE**

Introduction

Thank you for choosing to participate in this research study considering the assessment of hiv discordance and associated factors among sexual partners receiving HIV test in Dilla, Ethiopia. The following questionnaire is designed to collect some basic background information about you will aid in interpretation of the results. Please complete the questions as accurately and honestly as you can. If the question is unclear to you, feel free to ask me what is meant by the question. If there is a question that makes you uncomfortable you can choose not to respond to it or any other question. If you feel an uncomfortable emotional response as a result of the question on this questionnaire, please inform me at once and measures will be taken to reduce your discomfort immediately. Take as much time as you require completing the questionnaire.

Instructions: *Please answer all the questions below to the best of your knowledge. Where boxes are provided tick* [√] *the most appropriate one*

| Participant No | Background information |  |
| --- | --- | --- |
| 101 | Age of participant | 1. _______ Years.  88. Don’t know ❐  99. No response ❐ |
| 102 | Sex | 1. Male ❐  2. Female ❐ |
| 103 | Marital status | 1. Married ❐  2. Premarital ❐  3. Pre sexual ❐  4. Sex partner ❐  5. Others ______________  6. Nosponse ❐ |
| 104 | Educational status | 1. Illiterate ❐  2. Able to read ❐  3. Primary (1-8) ❐  4. Secondary (9-10) ❐  5.preparatory (11-12) ❐  6. Tertiary (college/university) ❐  7. Other (specify) ______  99. No response ❐ |
| 105 | Your title | 1. Nurse   2. Other (specify) ______  99. No response ❐ |
| 106 | How many years have you served as HIV counsellor? | 1. ______ Years  99. No response |
| 107 | Religion | 1. Orthodox ❐  2. Catholic ❐  3. Muslim ❐  4. Protestant ❐  5. No religion ❐  6. Other (specify)_________  99. No response ❐ |
| 108 | Your Monthly income | 1.___________ birr  3.No response ❐ |

**Part1 ፡ General** information about study participants

| **Participant** | **Age** | **Sex** | **Marital status** | **Educational status** | **Religion** | **Job status** | **Remark** |
| --- | --- | --- | --- | --- | --- | --- | --- |
| **1** |  |  |  |  |  |  |  |
| **2** |  |  |  |  |  |  |  |
| **3** |  |  |  |  |  |  |  |
| **4** |  |  |  |  |  |  |  |
| **5** |  |  |  |  |  |  |  |
| **6** |  |  |  |  |  |  |  |
| **6** |  |  |  |  |  |  |  |
| **7** |  |  |  |  |  |  |  |
| **8** |  |  |  |  |  |  |  |
| **9** |  |  |  |  |  |  |  |
| **10** |  |  |  |  |  |  |  |
| **11** |  |  |  |  |  |  |  |

**Tool 04**

**PART II: ACCESS TO SERVICE PROVISION**

| **201. Have you given any form of counselling on Sexual and Reproductive Health & Rights with emphasis on discordant relationship?**  1. Yes ❐  2. No ❐ | **202. Do sero-discordant couples’ have necessary information to determine the number, timing and spacing of their children?**  1. Strongly disagree ❐  2. Somewhat disagree ❐  3. Neither disagree nor agree ❐  4. Somewhat agree ❐  5. Strongly agree ❐ | **203. Do you offer a non-judgmental, supportive environment in which sero-discordant couples can share in confidence their challenges and experiences in SRH?**  1. Strongly disagree ❐  2. Somewhat disagree ❐  3. Neither disagree nor agree ❐  4. Somewhat agree ❐  5. Strongly agree ❐ |
| --- | --- | --- |
| **204. Do you offer prevention strategies so that sero-discordant couples are provided with necessary information and support to make informed decisions about their sexual health and that of their sexual partner(s)?**  1. Yes ❐  2. No ❐ | **205. Do you offer sero–discordant couples’ information about safer sex practices based on principles of prevention?**  1. Strongly disagree ❐  2. Somewhat disagree ❐  3. Neither disagree nor agree ❐  4. Somewhat agree ❐  5. Strongly agree ❐ | **206. Do you offer information that acknowledges positive aspects of sexuality, including aspects of safer sex that focus on attaining sexual pleasure and satisfaction to sero -discordant couples?**  1. Strongly disagree ❐  2. Somewhat disagree ❐  3. Neither disagree nor agree ❐  4. Somewhat agree ❐  5. Strongly agree ❐ |
| **207. Do you offer information and counseling on fertility options including information on infertility services, advice on planning a pregnancy and contraception to sero-discordant clients?**  1. Strongly disagree ❐  2. Somewhat disagree ❐  3. Neither disagree nor agree ❐  4. Somewhat agree ❐  5. Strongly agree ❐ | **208. Do yous promote and provide female clients in sero-discordant relationship with access to the female condom?**  1. Strongly disagree ❐  2. Somewhat disagree ❐  3. Neither disagree nor agree ❐  4. Somewhat agree ❐  5. Strongly agree ❐ | **209. Do you offer information about possibilities to advocate for realization of sexual and reproductive health rights of sero –discordant couples’?**  *For example, in accessing SRH services that are sensitive to the specific SRH needs in terms of being in a sero--‐discordant sexual relationship, deciding whether or not to have children.*  1. Yes ❐  2. No ❐ |
| **210. Do you offer information about and referrals to PMTCT for women in sero-discordant relationship who wish to get pregnant or women who are already pregnant?**  1. Strongly disagree ❐  2. Somewhat disagree ❐  3. Neither disagree nor agree ❐  4. Somewhat agree ❐  5. Strongly agree ❐ | **211. Do you offer PMTCT sero-discordant clients information about the risk and benefits of different modes of delivery, and inform them about their access to elective caesarean section?**  1. Strongly disagree ❐  2. Somewhat disagree ❐  3. Neither disagree nor agree ❐  4. Somewhat agree ❐  5. Strongly agree ❐ | **212. Are sero-discordant clients offered information and access (either directly or via a referral to another facility) to other contraceptive methods such as hormonal and injectable contraceptives, a diaphragm or an IUD in service provision centres?**  1. Strongly disagree ❐  2. Somewhat disagree ❐  3. Neither disagree nor agree ❐  4. Somewhat agree ❐  5. Strongly agree ❐ |
| **213. Do health care centres provide a supportive environment in which sero-discordant couples can share SRH experiences, needs and desires and discuss in a sensitive, non-judgmental and inclusive manner?**  1. Strongly disagree ❐  2. Somewhat disagree ❐  3. Neither disagree nor agree ❐  4. Somewhat agree ❐  5. Strongly agree ❐ | **214. 219. Are you aware of any integrated HIV/Sexual and reproductive health facility?**  1. Yes ❐  2. No ❐  If yes, state where…………..... | **215. Do health care centres offer sero-discordant clients with an appropriate referral for routine cervical screening and other sexual and reproductive health concerns?**  1. Strongly disagree ❐  2. Somewhat disagree ❐  3. Neither disagree nor agree ❐  4. Somewhat agree ❐  5. Strongly agree ❐ |
| **216. Do service providers’ and health centres offer information on the legal, medical and counselling support services available to women in the event of sexual and gender based violence in sero-discordant relationship?**  1. Strongly disagree ❐  2. Somewhat disagree ❐  3. Neither disagree nor agree ❐  4. Somewhat agree ❐  5. Strongly agree ❐ | **217. Do service providers have the requisite skills to handle sero-discordant couples sexual and reproductive health needs?**  1. Strongly disagree ❐  2. Somewhat disagree ❐  3. Neither disagree nor agree ❐  4. Somewhat agree ❐  5. Strongly agree ❐ | **218. Are our health facilities well prepared to manage SRH needs of sero-discordant couples?**  1. Strongly disagree ❐  2. Somewhat disagree ❐  3. Neither disagree nor agree ❐  4. Somewhat agree ❐  5. Strongly agree ❐ |

**Tool 04**

PART III: INTERVIEW GUIDE

| **No 301** | **Objective: To explore knowledge on HIV discordance**  **Q1. Do you have any question before we begin?**  **Probe:**   1. **Can one partner be HIV negative if the other partner is HIV positive?** 2. **How do you explain/define HIV discordance?** 3. **Why HIV discordance exists? How?** 4. **What are your sources of information about discordance?** 5. **How HIV- negative discordant couple is actually infected? Why?**   **Q2. Did you know how HIV transmission is prevented?**  **Probe:**   1. **If both HIV positive, does this mean that one partner infected the other?** 2. **If a partner is HIV positive, is it possible to prevent transmission to HIV-negative person?** 3. **If a partner is HIV positive and other is HIV negative, does this mean HIV-positive partner has been unfaithful?** 4. **Which type sex is related with risk of HIV transmission? Why?** 5. **How long God’s protection would last? Why?**   **Q3. Did you give Couple HIV counselling and testing? Yes ❐ No ❐**  **Probe:**   1. **Do you think it was helpful? Explain your answer** 2. **How did you handle the situation?** 3. **Have you given any form of counselling on Sexual and Reproductive Health& Rights?** |
| --- | --- |
| **302** | **Objective: To explore the challenges of HIV discordance**  **Probe :**   1. **Which health needs do you think is most important to HIV discordant couples?** 2. **What is your experience about local services such as counsellor, health facility etc?** 3. **What kind of challenges did face in their sexual relations?** 4. **What type of advices was given by you to have children for HIV discordant couples?** |
| **303** | **Objective: To explore the implication of desire of children on HIV transmission of HIV**  **Q4. What kind of ways do you use to have child?**  **Probe:**   1. **What will happen if they practice unsafe sex to have children? How?** 2. **What recommendations do you have for future efforts such as these?** 3. **Can you think of the impact of HIV discordance? Explain** |
| **304** | **Objective: To explore on coping choices by HIV discordant couples**  **Q5. What has helped them most to cope?**  ***Probe:***   1. **What should counsellor offer?** 2. **What kind of preventive strategies do you suggest to prevent HIV transmission? How?**   **Q6. What kind of behaviour encourages HIV transmission risk among discordant couple?**  **Probe:**   1. **What kind of behaviour encourages risk reduction among discordant couple? Why? Explain** 2. **What are your suggestions for discordant couple interventions?** 3. **What are your choices on Sexual and reproductive health and reproductive choices?** 4. **Is there anything else that we should have talked about that we did not?** |

**Thank you for taking time to fill in this questionnaire.**

**God richly bless you!**
