# Supplementary material for: Assessment of HIV discordance and associated risk factors among couples receiving HIV test in Dilla, Ethiopia
Source: BMC Res Notes. 2014 Dec 10;7:893. doi: 10.1186/1756-0500-7-893 (PMC4295257; doi:10.1186/1756-0500-7-893)
Supplement: Supplementary file 5 — Additional file 5: FGD questioner for exploring the paradox from discordant couples. (DOC 92 KB) [file 13104_2012_3387_MOESM5_ESM.doc]

Tool 05

Schedule No: _____

**A FGD GUIDE FOR EXPLORING THE PARADOX: KNOWLEDGE, CHALLENGES, AND COPING CHOICES AMONG HIV DISCORDANT COUPLES IN DILLA TOWN**

Code of Respondent: ...................................................................

Name of Interviewer/ Counsellor: ...................................................................

Code of VCT centre/health facility/Location of Interview: ..................................................................

Date of Interview: ...................................................................

Time of Interview: ...................................................................

Instructions: *Please answer all the questions below to the best of your knowledge.*

**How to respond the questionnaire**

1. This questionnaire has 3 parts.
2. Each question has its own serial number, question, and answer
3. Part 1. 6 questions, and
4. Part 2. 18 questions
5. Part 2. 4 objectives questions

**Site (VCT center) type**

Integrated ❐

Free standing ❐

NGO ❐

Youth ❐

Mobile ❐

Private ❐

Home-based ❐

Work place ❐

Governmental ❐

Other (specify) ______________________________

**Tool 05**

**A FGD GUIDE FOR EXPLORING THE PARADOX: KNOWLEDGE. CHALLEGES, AND COPING CHOICES AMONG HIV DISCORDANT COUPLES**

**CONSENT FORM**

Hello my name is _______________________ and I work for an organization named Dilla university school of health sciences found in Dilla town. I am here to collect information for the research to be conducted on HIV discordance and associated factors. The purpose of the study is to understand the associated factors with occurrence of HIV discordance among sexual partners and establish evidence and support the activities carried out to posive prevention strategies in Dilla town as well as in the country. The questionnaire will take 20-30 minutes.

In the questionnaire you’ll be asked some very personal questions that some people find it difficult to answer. Your name will not be written on this questionnaire, and will never be used in connection with any of the information you tell me. You are selected for this survey merely by chance, not done intentionally.

Participation is based on your willingness besides; you can withdraw from the study anytime. However your kin participation would be very useful. In addition, no personal identification will be written and we assure you that what ever information you are providing will only be used for the research purpose and the data will be handled only by the research team. While we are collecting the data it is difficult to jot down everything thus we will tape record our discussion.

**Participant’s statement**

I know what this research study is about and I know what will do if choose to take part. I have had a chance to ask question and I know I can ask question at any time during or after the interview. I know I am free to not answer a question or quit at any time. I freely choose to be a part of this study. If you need any further information about the study please contact the following person.

Moges Tadesse

Dilla University, school of health sciences

Tel: 0911923244

Are you willing to participate in the study?

Agreed __________

Not Agreed ____________

Thank you for your time and contribution.

Name of Data collector ___________________ signature ______________________

Date of data collection _____________

**Tool 05**

**PART I : GENERAL INFORMATION**

**DEMOGRAPHIC QUESTIONNAIRE**

Introduction

Thank you for choosing to participate in this research study considering the assessment of hiv discordance and associated factors among sexual partners receiving HIV test in Dilla, Ethiopia. The following questionnaire is designed to collect some basic background information about you will aid in interpretation of the results. Please complete the questions as accurately and honestly as you can. If the question is unclear to you, feel free to ask me what is meant by the question. If there is a question that makes you uncomfortable you can choose not to respond to it or any other question. If you feel an uncomfortable emotional response as a result of the question on this questionnaire, please inform me at once and measures will be taken to reduce your discomfort immediately. Take as much time as you require completing the questionnaire.

Instructions: *Please answer all the questions below to the best of your knowledge. Where boxes are provided tick* [√] *the most appropriate one*

| Participant No | Background information |  |
| --- | --- | --- |
| 101 | Age of participant | 1. _______ Years.  88. Don’t know ❐  99. No response ❐ |
| 102 | Sex | 1. Male ❐  2. Female ❐ |
| 103 | Marital status | 1. Married ❐  2. Premarital ❐  3. Pre sexual ❐  4. Sex partner ❐  5. Others ______________  6. Nosponse ❐ |
| 104 | Educational status | 1. Illiterate ❐  2. Able to read ❐  3. Primary (1-8) ❐  4. Secondary (9-10) ❐  5.preparatory (11-12) ❐  6. Tertiary (college/university) ❐  7. Other (specify) ______  99. No response ❐ |
| 105 | Your title | 1. Nurse   2. Other (specify) ______  99. No response ❐ |
| 106 | How many years have you served as HIV counsellor? | 1. ______ Years  99. No response |
| 107 | Religion | 1. Orthodox ❐  2. Catholic ❐  3. Muslim ❐  4. Protestant ❐  5. No religion ❐  6. Other (specify)_________  99. No response ❐ |
| 108 | Your Monthly income | 1.___________ birr  3.No response ❐ |

**Part1 ፡ General** information about study participants

| **Participant** | **Age** | **Sex** | **Marital status** | **Educational status** | **Religion** | **Job status** | **Remark** |
| --- | --- | --- | --- | --- | --- | --- | --- |
| **1** |  |  |  |  |  |  |  |
| **2** |  |  |  |  |  |  |  |
| **3** |  |  |  |  |  |  |  |
| **4** |  |  |  |  |  |  |  |
| **5** |  |  |  |  |  |  |  |
| **6** |  |  |  |  |  |  |  |
| **6** |  |  |  |  |  |  |  |
| **7** |  |  |  |  |  |  |  |
| **8** |  |  |  |  |  |  |  |
| **9** |  |  |  |  |  |  |  |
| **10** |  |  |  |  |  |  |  |
| **11** |  |  |  |  |  |  |  |

**Tool 05**

**PART II: FGD GUIDE**

| **No 201** | 1. **Objective: To explore knowledge on HIV discordance**   **Q1. Do you have any question before we begin?**  **Probe:**   - Can one partner be HIV negative if the other partner is HIV positive? - Define HIV discordance? - Why HIV discordance exists? How?   **Q2. Did you know how HIV transmission is prevented?**  **Probe:**   - If both HIV positive, does this mean that one partner infected the other? - If a partner is HIV positive and other is HIV negative, does this mean HIV-positive partner has been unfaithful? - Which type sex is related with risk of HIV transmission? Why? |
| --- | --- |
| **No 202** | 1. **Objective: To explore the challenges of HIV discordance**   **Q3. What kind of person do you choose to disclose your HIV status?**  **Probe:**   - Which health needs do you think is most important to HIV discordant couples? - What kind of challenges did face in your sexual relations? - Describe challenges associated with your discordant status? - What type of advices was given to you to have children from health providers? - Do you have Challenges in practising safer sex in your sero discordant relationship? - Difficulties in Disclosure of your status? Explain   **Q4. Do you have experiences of stigma and discrimination?**  **Probe:**   - What happened then? - Rejection of HIV-positive partner by current or former partners - Experiences of domestic violence - How does that affect your health?   **Q5. Did you give Couple HIV counselling and testing? Yes ❐ No ❐**  **Probe:**   - Do you think it was helpful? Explain your answer - How did you handle the situation? - Have you given any form of counselling on Sexual and Reproductive Health& Rights? - Which health needs do you think is most important to HIV discordant couples? - What is your experience about local services such as counsellor, health facility etc? - What kind of challenges did face in their sexual relations? - What type of advices was given by you to have children for HIV discordant couples?   **Q6. How long have you been in a sero-discordant relationship and elaborate on your wellness status and that of your partner?**  **Q7 Have you experienced any form of tension in your relationship as a result of you have been in a discordant relationship? Explain**  **Q8. As an individual, do you decide freely and responsibly the number, timing and spacing of children without interference? Explain**  **Probe**   - Need to have children/desire? Why? - Discussion with partner about number of children to have - When to have children - Had children after knowing status? - If Yes, why? - What is the implication of desire of children on HIV transmission of HIV   **Q9. Do you have the information and education and means to achieve question 9.above? Explain**  **Q10. Have you discussed with health provider/counsellor about**  **probe**   - Contraception - Pregnancy and HIV - Birth spacing and HIV - Abortion discussed - Condom use - Currently using contraception - If you are or partner pregnant? Why?   **Q11. Do you have adequate and accurate information on how to handle your sexual needs in order to attain the highest standard of sexual and reproductive health? Explain**  **Q12. What are the safer sex practices you have adopted with your partner? Explain**  **Q13. Frequency of you ever had sex with your partner**  **Q14. What can you tell me about Condom use during sexual intercourse?**  **Q15. Integrated HIV/Sexual and reproductive health facility to HIV discordant couple**  **Probe**   1. **Have you and your partner received any form of counselling on Sexual and Reproductive Health& Rights (SRH&R)?**   **PROBE**   - To determine the number, timing and spacing of your children. Explain - Is it non-judgmental, supportive environment to share in confidence your challenges and experiences in SRH? Explain  1. **Do service providers offer prevention strategies?**   **PROBE**   - Information and support to make informed decisions about your sexual health and that of your sexual partner(s)? explain  1. **Do service providers’ offer information about safer sex practices?**   **PROBE**   - Aspects of safer sex that focus on attaining sexual pleasure and satisfaction. explain   Positive aspects of sexuality. Explain   1. **Do service providers’ offer information and counselling on fertility options?**   **PROBE**   - Infertility services. Explain - Advice on planning a pregnancy. explain. - Contraception, explain - Promote and provide female clients in sero-discordant relationship with access to the female condom explain. - Advocate for the realization of the sexual and reproductive health rights of sero –discordant couples’? explain - Referrals to PMTCT for women in sero-discordant relationship who wish to get pregnant or women who are already pregnant? Why? - Risk and benefits of different modes of delivery, and inform you about your access to elective caesarean section? - Information and access (either directly or via a referral to another facility) to other contraceptive methods such as hormonal and injectable contraceptives, a diaphragm or an IUD in service provision centres? Explain - Provide a supportive environment in which sero-discordant couples can share SRH experiences, needs and desires and discuss in a sensitive, non-judgmental and inclusive manner? Explain - An appropriate referral for routine cervical screening and other sexual and reproductive health concerns? Why? - Information on the legal, medical and counselling support services available to women in the event of sexual and gender based violence in sero-discordant relationship? Explain  1. **Do service providers have the requisite skills to handle sero-discordant couples sexual and reproductive health needs? Explain** 2. **Are these health facilities well prepared to manage the SRH needs of sero-discordant couples? Explain**   **Are you aware of any integrated HIV/Sexual and reproductive health facility? Explain** |
| **No 203** | 1. **Objective: To explore on coping choices by HIV discordant couples**   **Q16. What has helped them most to cope?**  ***Probe:***   - What should counsellor offer? - What kind of preventive strategies do you suggest to prevent HIV transmission? How?   **Q17. What kind of behaviour encourages HIV transmission risk among discordant couple?**  **Probe:**   - What kind of behaviour encourages risk reduction among discordant couple? Why? Explain - What are your suggestions for discordant couple interventions? - What are your choices on Sexual and reproductive health and reproductive choices?   Is there anything else that we should have talked about that we did not? |

**Thank you for taking time to fill in this questionnaire.**

**God richly bless you!**
