# Supplementary material for: Assessment of HIV discordance and associated risk factors among couples receiving HIV test in Dilla, Ethiopia
Source: BMC Res Notes. 2014 Dec 10;7:893. doi: 10.1186/1756-0500-7-893 (PMC4295257; doi:10.1186/1756-0500-7-893)
Supplement: Supplementary file 6 — Additional file 6: FGD questioner for exploring the paradox from counsellors. (DOC 88 KB) [file 13104_2012_3387_MOESM6_ESM.doc]

Tool 06

Schedule No: _____

**A FGD GUIDE FOR EXPLORING THE PARADOX: KNOWLEDGE, CHALLENGES, AND COPING CHOICES AMONG HIV DISCORDNAT COUPLES IN DILLA TOWN**

Code of Interviewee/ Counsellor: ...................................................................

Code of VCT centre/health facility/Location of Interview: ..................................................................

Date of Interview: ...................................................................

Time of Interview: ...................................................................

Instructions: *Please answer all the questions below to the best of your knowledge.*

**How to respond the questionnaire**

1. This questionnaire has 2 parts.
2. Each question has its own serial number, question, and answer
3. Part 1: 8 questions, and
4. Part 2: 3 objectives questions

**Site (VCT centre) type**

Integrated ❐

Free standing ❐

NGO ❐

Youth ❐

Mobile ❐

Private ❐

Home-based ❐

Work place ❐

Governmental ❐

Other (specify) ______________________________

**Tool 06**

**A FGD GUIDE FOR EXPLORING THE PARADOX: KNOWLEDGE, CHALLEGES, AND COPING CHOICES AMONG HIV DISCORDANT COUPLES**

**CONSENT FORM**

Hello my name is _______________________ and I work for an organization named Dilla university school of health sciences found in Dilla town. I am here to collect information for the research to be conducted on exploring the paradox: knowledge, Challenges and coping choices among HIV discordant couples. The purpose of the study is to explore the paradox: knowledge, Challenges and coping choices among HIV discordant couples and establish evidence and support the activities carried out to positive prevention strategies in Dilla town as well as in the country. The questionnaire will take 20-30 minutes.

In the questionnaire you’ll be asked some very personal questions that some people find it difficult to answer. Your name will not be written on this questionnaire, and will never be used in connection with any of the information you tell me. You are selected for this survey merely by chance, not done intentionally.

Participation is based on your willingness besides; you can withdraw from the study anytime. However your kin participation would be very useful. In addition, no personal identification will be written and we assure you that what ever information you are providing will only be used for the research purpose and the data will be handled only by the research team. While we are collecting the data it is difficult to jot down everything thus we will tape record our discussion.

**Participant’s statement**

I know what this research study is about and I know what will do if choose to take part. I have had a chance to ask question and I know I can ask question at any time during or after the interview. I know I am free to not answer a question or quit at any time. I freely choose to be a part of this study. If you need any further information about the study please contact the following person.

Moges Tadesse

Dilla University, school of health sciences

Tel: 0911923244

Are you willing to participate in the study?

Agreed __________

Not Agreed ____________

Thank you for your time and contribution.

Name of Data collector ___________________ signature ______________________

Date of data collection _____________

**Tool 06**

**PART I : GENERAL INFORMATION**

**DEMOGRAPHIC QUESTIONNAIRE**

Introduction

Thank you for choosing to participate in this research study considering the exploring the paradox: knowledge, challenges, and coping choices among HIV discordant couples in Dilla, Ethiopia. The following questionnaire is designed to collect some basic background information about you will aid in interpretation of the results. Please complete the questions as accurately and honestly as you can. If the question is unclear to you, feel free to ask me what is meant by the question. If there is a question that makes you uncomfortable you can choose not to respond to it or any other question. If you feel an uncomfortable emotional response as a result of the question on this questionnaire, please inform me at once and measures will be taken to reduce your discomfort immediately. Take as much time as you require completing the questionnaire.

Instructions: *Please answer all the questions below to the best of your knowledge. Where boxes are provided tick* [√] *the most appropriate one*

| Participant No | Background information |  |
| --- | --- | --- |
| 101 | Age of participant | 1. _______ Years.  88. Don’t know ❐  99. No response ❐ |
| 102 | Sex | 1. Male ❐  2. Female ❐ |
| 103 | Marital status | 1. Married ❐  2. Premarital ❐  3. Pre sexual ❐  4. Sex partner ❐  5. Others ______________  6. Nosponse ❐ |
| 104 | Educational status | 1. Illiterate ❐  2. Able to read ❐  3. Primary (1-8) ❐  4. Secondary (9-10) ❐  5.preparatory (11-12) ❐  6. Tertiary (college/university) ❐  7. Other (specify) ______  99. No response ❐ |
| 105 | Your title | 1. Nurse   2. Other (specify) ______  99. No response ❐ |
| 106 | How many years have you served as HIV counsellor? | 1. ______ Years  99. No response |
| 107 | Religion | 1. Orthodox ❐  2. Catholic ❐  3. Muslim ❐  4. Protestant ❐  5. No religion ❐  6. Other (specify)_________  99. No response ❐ |
| 108 | Your Monthly income | 1.___________ birr  3.No response ❐ |

**Part1 ፡ General** information about study participants

| **Participant** | **Age** | **Sex** | **Marital status** | **Educational status** | **Religion** | **Job status** | **Remark** |
| --- | --- | --- | --- | --- | --- | --- | --- |
| **1** |  |  |  |  |  |  |  |
| **2** |  |  |  |  |  |  |  |
| **3** |  |  |  |  |  |  |  |
| **4** |  |  |  |  |  |  |  |
| **5** |  |  |  |  |  |  |  |
| **6** |  |  |  |  |  |  |  |
| **6** |  |  |  |  |  |  |  |
| **7** |  |  |  |  |  |  |  |
| **8** |  |  |  |  |  |  |  |
| **9** |  |  |  |  |  |  |  |
| **10** |  |  |  |  |  |  |  |
| **11** |  |  |  |  |  |  |  |

**Tool 05**

**PART II: FGD GUIDE**

| **No 201** | 1. **Objective: To explore knowledge on HIV discordance**   **Q1. Do you have any question before we begin?**  **Probe:**   - 1. Can one partner be HIV negative if the other partner is HIV positive?   2. Define HIV discordance?   3. Why HIV discordance exists? How?   **Q2. Did you know how HIV transmission is prevented?**  **Probe:**   - 1. If both HIV positive, does this mean that one partner infected the other?   2. If a partner is HIV positive and other is HIV negative, does this mean HIV-positive partner has been unfaithful?   3. Which type sex is related with risk of HIV transmission? Why? |
| --- | --- |
| **No 202** | 1. **Objective: To explore the challenges of HIV discordance**   **Q3. Which health needs do you think is most important to HIV discordant couples?**  **Probe:**   - 1. What type of advices should be given to them to have children?   2. What is the implication of desire of children on HIV transmission of HIV   **Q4. Do you give Couple HIV counselling and testing? Yes ❐ No ❐**  **Probe:**   - 1. Do you think it was helpful? Explain your answer   2. How did you handle the HIV discordance?   3. What type of counselling on Sexual and Reproductive Health& Rights should be given?   **Q5. Have you discussed with your clients(HIV discordant couples) about**  **probe**   - 1. Contraception, explain   2. Pregnancy and HIV   3. Birth spacing and HIV   4. Abortion discussed   5. Condom use   6. Currently using contraception   7. If client is or partner pregnant, What should be done? Why?   **Q6. Do you think that they have adequate and accurate information on how to handle their sexual and reproductive health needs? Explain**  **Probe**   - 1. Infertility services. Explain   2. Advice on planning a pregnancy. explain.   3. Access to the female condom, explain.   4. Referrals to PMTCT for women in sero-discordant relationship who wish to get pregnant or women who are already pregnant? Why?   5. Risk and benefits of different modes of delivery, and access to elective caesarean section?   6. Access to other contraceptive methods such as hormonal and injectable contraceptives, a diaphragm or an IUD?   7. An appropriate referral for routine cervical screening and other sexual and reproductive health concerns? Why?   8. Information on the legal, medical and counselling support services available to women in the event of sexual and gender based violence in sero-discordant relationship? Explain   **Q7. Do you think that you have the requisite skills to handle sero-discordant couples sexual and reproductive health needs? Explain**  **Q8. Are these health facilities in which you are working well prepared to manage the SRH needs of sero-discordant couples? Explain**  **Q9. Are you trained on integrated HIV/Sexual and reproductive health? Explain** |
| **No 203** | 1. **Objective: To explore on coping choices by HIV discordant couples**   **Q10. What has helped them most to cope?**  ***Probe:***   - 1. What should counsellor offer?   2. What kind of preventive strategies do you suggest to prevent HIV transmission? How?   3. What are your suggestions for discordant couple interventions?   4. What are your choices on Sexual and reproductive health and reproductive choices?   5. Is there anything else that we should have talked about that we did not? |

**Thank you for taking time to fill in this questionnaire.**

**God richly bless you!**
